# Understanding resilience in same-sex parented families: the work, love, play study

**DOI:** 10.1186/1471-2458-10-115

**Published:** 2010-03-09

**Authors:** Jennifer J Power, Amaryll Perlesz, Margot J Schofield, Marian K Pitts, Rhonda Brown, Ruth McNair, Anna Barrett, Andrew Bickerdike

**Affiliations:** 1 The Bouverie Centre, La Trobe University, 8 Gardiner Street, Brunswick, Victoria 3056, Australia; 2 School of Public Health, La Trobe University, Melbourne, Victoria 3086, Australia; 3 Australian Research Centre in Sex, Health and Society, LaTrobe University, 215 Franklin St, Victoria 3000, Australia; 4 School of Nursing, Deakin University, 221 Burwood Highway, Burwood, Victoria, 3125, Australia; 5 Department of General Practice, University of Melbourne, 200 Berkeley Street, Carlton, Victoria 3053, Australia; 6 Relationships Australia Victoria, 46 Princess Street, Kew, Victoria 3101, Australia

## Abstract

**Background:**

While families headed by same-sex couples have achieved greater public visibility in recent years, there are still many challenges for these families in dealing with legal and community contexts that are not supportive of same-sex relationships. The Work, Love, Play study is a large longitudinal study of same-sex parents. It aims to investigate many facets of family life among this sample and examine how they change over time. The study focuses specifically on two key areas missing from the current literature: factors supporting resilience in same-sex parented families; and health and wellbeing outcomes for same-sex couples who undergo separation, including the negotiation of shared parenting arrangements post-separation. The current paper aims to provide a comprehensive overview of the design and methods of this longitudinal study and discuss its significance.

**Methods/Design:**

The Work, Love, Play study is a mixed design, three wave, longitudinal cohort study of same-sex attracted parents. The sample includes lesbian, gay, bisexual and transgender parents in Australia and New Zealand (including single parents within these categories) caring for any children under the age of 18 years. The study will be conducted over six years from 2008 to 2014. Quantitative data are to be collected via three on-line surveys in 2008, 2010 and 2012 from the cohort of parents recruited in Wave1. Qualitative data will be collected via interviews with purposively selected subsamples in 2012 and 2013. Data collection began in 2008 and 355 respondents to Wave One of the study have agreed to participate in future surveys. Work is currently underway to increase this sample size. The methods and survey instruments are described.

**Discussion:**

This study will make an important contribution to the existing research on same-sex parented families. Strengths of the study design include the longitudinal method, which will allow understanding of changes over time within internal family relationships and social supports. Further, the mixed method design enables triangulation of qualitative and quantitative data. A broad recruitment strategy has already enabled a large sample size with the inclusion of both gay men and lesbians.

## Background

Families headed by same-sex couples have achieved greater public visibility in the western world in recent years, with increasing media and political attention being devoted to debates on gay marriage [1,2] and the reproductive rights of lesbians and gay men, including the right to access in vitro-fertilisation (IVF) and surrogacy [3,4]. Accompanying this is a growing body of academic research that has sought to increase sociological and psychological understanding of the experiences of lesbian, gay, bisexual and transgender (GLBT) parents and their children [5-12].

It is difficult to estimate accurately the number of families headed by same-sex attracted parents in any country [4,13]. Data from the 2001 Australian census indicates that one in five lesbian couples, and up to five percent of gay male couples, have a child or children living with them at home. This figure is likely to considerably underestimate the actual number of same-sex attracted parents as it does not include single parents and parents who have children not living at home with them [14]. Similarly, the US Census Bureau in 2000 estimated that one-third of lesbian and one-fifth of gay male headed households were currently raising children [15]. Irrespective of actual figures, however, it is clear that a substantial number of lesbians and gay men are likely to become parents at some point in their lives.

Research shows that same-sex couples and same-sex attracted sole parents form their families in numerous ways. A large number of lesbians and gay men have children from previous heterosexual relationships [4]. As well, many lesbian-couples conceive their children within their relationship using anonymous sperm donors accessed through fertility clinics [4,6,16,17] or a known donor who may or may not become part of their child's life or be known as a father to the child [2,5,18]. Donating sperm to lesbians provides an opportunity for many gay men to become parents, some of whom will have ongoing co-parenting/shared-care arrangements with the children's mother/s [2,4,5,18,19]. A smaller, but increasing, number of gay men have become parents through surrogacy arrangements [2,4]. Lesbians and gay men may also foster or adopt children in contexts where this is legally permitted [2,13,20]. Many same-sex parents have blended families that include children from previous relationships as well as children conceived within the relationship by one or both of the parents[5]. This diversity of family forms challenges normative, heterosexually-based, concepts or definitions of family[18].

The biggest challenge for most same-sex parented families is dealing with a legal and community context that is generally not supportive of same-sex relationships and often does not accommodate diverse family forms [1,6,15]. Normative concepts of family centre on the assumption of biological ties between children and their heterosexual parents [21]. In same-sex relationships, non-biological parents of children conceived within the relationship often have few means to ensure they are legally recognised as a parent [4]. It is also possible that extended families may not acknowledge non-biological children or parents as part of their family [4].

Same-sex parented families may experience discrimination within the health, welfare, education and legal systems [4,8,22-24] or encounter negative attitudes by service providers when negotiating critical life events such as becoming pregnant, dealing with pre and post-natal services, arranging child care or sending children to school [22,25]. Even where there is no overt discrimination, engagement with formal systems such as childcare facilities, schools and the health system, regularly requires same-sex parented families to negotiate processes designed with the assumption that all households are headed by a heterosexual couple. For instance, most enrolment or information forms relating to a child do not have provisions for two mothers or two fathers. This means same-sex parents regularly have to negotiate with service providers to ensure both the child's parents are acknowledged [2,24]. For some parents, the process of having to 'out' themselves on regular occasions can be stressful [20,24] and many worry about their children experiencing homophobic harassment or discrimination at school [20,22,24].

On a more personal level, some lesbian and gay parents encounter a lack of support, or in some cases outright hostility, within their own extended family network [5,24,26,27]. Added to this, when lesbians or gay men become parents they may lose some of their connections within the lesbian and gay community, an important source of support and validation for many same-sex attracted individuals [24].

Considerable research shows that people who are same-sex attracted experience marginalisation and stigmatisation as a result of their sexual identity [28]. This potentially contributes to reduced social connectedness, and can have a negative impact on same-sex attracted people's physical and mental health and access to health care [28,29]. This may be compounded for same-sex attracted parents who feel unsupported and experience psychological distress related to their concerns about the impact of homophobia on their children [9]. Shapiro et al (2009) conducted a study comparing the psychological wellbeing of lesbian and heterosexual mothers in Canada (where same-sex marriage is legal and there are numerous provisions protecting the rights of same-sex parents) and the United States (where same-sex partnerships receive little legal protection in most states). The study found that sexual orientation in itself does not contribute to poorer mental health among lesbian mothers, but the legal and social context in which they parent does. Lesbian mothers in the United States expressed more worry about their legal status as a parent and showed more depressive symptoms than lesbian mothers in Canada [15].

Despite these challenges, there is a large body of research that indicates the developmental, social and emotional outcomes for children raised in same-sex parented families are at least equal to those of their heterosexual-parented peers[30]. The studies suggest that it is processes within the family - including quality of relationships and the psychosocial well-being of parents - that contribute to higher levels of wellbeing among children, irrespective of parents' sexual orientation [4,6,7,13,30-38]. Furthermore, recent studies have shown that lesbian mothers tend to organise family and work responsibilities more equitably than heterosexual couples, which may contribute to higher levels of relationship satisfaction [4,10,30,39].

However, there are some gaps in the research on same-sex parented families. Firstly, the dominance of research focusing on outcomes for children raised in same-sex parented families means there is only a limited number of studies on the health and wellbeing of same-sex attracted parents. There is also little research on factors that support and enhance resilience in same-sex headed families [13]. What enables one family to cope in stressful situations while others struggle?

A further gap in the research relates to same-sex attracted parents' experiences of separation. While it is well documented that at least one in three Australian heterosexual marriages ends in divorce [40], there has been very limited Australian research on separation rates for same-sex couples with children or the experience and impact of separation on parents' and children's health and wellbeing. In one small Australian study of 25 lesbian-parented families, around 20% of couples had separated before the end of the study [41]. One international longitudinal study of lesbian mothers who had conceived children using donor insemination, found that 38% (n = 30) of couples had separated by the time their child was 10 years old [9]. However, research has not looked at whether separating couples are able to access appropriate support or at health and wellbeing outcomes.

The concept of resilience has been applied in a number of studies of heterosexual families [42-44]. Family resilience can be viewed as the extent to which families are able to balance stress and demands with the capabilities and strengths of that family unit - or "the presence of good outcomes despite adversity" [42,44]. When demands or stressors outweigh capabilities and resources, family crisis or breakdown may occur. A more resilient family will be able to harness its capabilities to successfully manage demands, risks and stressors [43].

Studying family resilience involves identification of factors that protect families from crisis or breakdown (separation). The literature on family resilience identifies these protective factors at three distinct, but integrated, levels: individual, family, and community [45]. Individual level protective factors include: higher levels of parental education and income, positive mental health indicators; strong coping skills and a general sense of optimism [42-44,46]. Family level protective factors include: cohesiveness of the family, quality of parental relationships (including quality of communication and negotiation of household and childcare responsibilities), and quality of relationships between parents and children [44,46,47]. Community level protective factors include: access to services and support, access to community networks, a sense of connection to the local community and to extended family, and strong social networks. At this level, formal institutional supports as well as informal networks and support systems are important [9,24,26,42-45]. In the case of same-sex parented families, legal recognition of relationships has also been shown to support resilience. The increased legitimacy that comes with legalisation enables the family to access more formal economic support and also opens opportunities for developing social connection [26,48,49].

There have been some studies which look at the resilience of gay and lesbian individuals or couples, but few have examined resilience in the context of same-sex parented families [26,49]. The Work, Love, Play (WLP) Study will examine the extent to which factors contributing to family resilience, as identified in previous research on heterosexual families, are associated with resilience within families where one or more parents identify as same-sex attracted and which may be more diverse in their structure than traditional heterosexual family structures. The WLP study will also identify factors that support family resilience and wellbeing in social contexts marked by legal and community heterosexism and discrimination towards same-sex parents and their children. The study will also look at the experience of separation among same-sex attracted couples who have children (see Table 1 for further description of the study aims). The aims of the WLP study are to:

**Table 1:** Research aims, relationship of aims to questionnaire topics/sections and benefit derived from this line of inquiry

Research aim	Topics/section in survey	Benefit
Describe the characteristics and diversity of same-sex parented families in Australia and New Zealand	Demographics Family formation Methods by which children were conceived	To increase knowledge of the way in which same-sex couples negotiate family structures that are more complex than in traditional families and the potential social, legal and health implications of this for same-sex parents.

Examine factors associated with relationship breakdown and resilience in same-sex parented families	Family formation (complexity of family structures) Engagement with local community and extended family Division of labour within the household Use of services Quality of parents' relationship Parents' health and wellbeing Legal status of parents' relationship	The longitudinal design of the study will enable us to follow changes over time in factors that support family resilience and make comparisons between those couples/families who separate and those who don't. This will help to identify areas where same-sex parented families can be better supported.

Examine changes in relationship stability over time and parenting arrangements among those who separate	Family formation (complexity of family structures) Quality of parents' relationship Engagement with local community and extended family Parents' health and wellbeing	Increase knowledge of whether same-sex parents receive formal and informal support post separation and the potential impact of separation on health and wellbeing of parents and children.

Explore the impact of discrimination and homophobic community attitudes on same-sex parented families	Use of services Engagement with local community and extended family Experiences of discrimination Parents' health and wellbeing	Increase knowledge about the relationship between experiences of discrimination and the health and wellbeing of same-sex parents; and whether discrimination isolates same-sex parents and their children from the mainstream community (including services).

• describe the characteristics and diversity of same-sex parented families in Australia and New Zealand

• examine factors associated with relationship breakdown and relationship resilience in same-sex parented families

• examine changes in relationship stability over time and parenting arrangements among those who separate

• explore the impact of discrimination and homophobic community attitudes on same-sex parented families

• identify barriers to appropriate service provision for same-sex parented families across a range of sectors including community and health services

• develop ways of translating research evidence on resilience in same-sex parented families into Good Practice Guidelines for use by providers in mainstream services and services targeting same-sex attracted individuals and families.

## Methods/Design

### Design

The WLP study was designed as a mixed method, three wave, longitudinal cohort study of same-sex attracted parents in Australia and New Zealand to be conducted over six years from 2008 to 2014. The volunteer sample comprises lesbian, gay, bisexual and transgender parents (including single parents within these categories) caring for any children under the age of 18 years. Quantitative data have been collected via the first on-line survey in 2008, with two further on-line surveys planned for 2010 and 2012. Qualitative data will be collected via interviews with purposively selected subsamples in 2012 and 2013 and through open-ended questions in the surveys. Key outcomes of interest for the sub-studies are: increasing knowledge of factors that contribute to resilience in same-sex parented families and increasing knowledge of same-sex attracted parents' experiences of partner separation and parenting arrangements post separation.

### Sample

The cohort sample will comprise volunteer participants from Australia and New Zealand who identify as same-sex attracted (including gay, lesbian, bisexual and transgender individuals) and who are currently actively engaged in parenting a child or children aged under 18 years. Sole-parents who identify as same-sex attracted are eligible to participate. The sampling strategy is limited to one respondent per family. The self-complete survey requires participants to speak and write English.

There is no adequate census data available to estimate the population size of same-sex attracted parents in Australia and New Zealand. Further, previous studies of this population group have involved only small samples and have been largely qualitative. As such, there is inadequate information to run a power calculation to determine an appropriate sample size for this study. Wave One data was collected between June and November 2008. In this period, 445 eligible people completed the survey, 355 (80%) of whom consented to take part in the longitudinal study. Further sampling and recruitment in specific categories began in 2009/early 2010 to augment the sample, aiming to include at least 50 cases in all categories of interest, including gender and place of residence.

Of the 445 respondents to the first round of Wave One, 85% (n = 377) resided in Australia and 15% (n = 68) in New Zealand. The majority identified as lesbian (75%, n = 334), with 15% (n = 65) identifying as gay, 8% (n = 36) as bisexual and 1% (3) as Takatäpui (a Māori term for same-sex attracted people) and 2% (n = 7) as "other", which included some transgender respondents. The majority of respondents (76%, n = 340) resided in inner or outer metropolitan areas. Just under one quarter of participants described their place of residence as either a regional centre or a rural/remote area (23%, n = 102). Of the 355 respondents who agreed to be contacted about participating in future surveys, 301 were women, 49 were men and four described their gender as "other".

Purposive subsamples of the cohort will be recruited from the Wave One cohort for qualitative interview studies around two specific areas of interest: factors supporting resiliency in same-sex parented families and the experience of parental separation in same-sex parented families.

### Recruitment

For the Wave One survey, volunteer participants were recruited via:

• a paid website banner appeared for three months on lesbian social networking site the 'Pink Sofa'

• advertisements in the Gay and Lesbian Health Victoria newsletter and website and the Brisbane "City-Lickers" lesbian news

• media releases sent to gay and lesbian press across Australia and New Zealand, from which at least three print-media articles or notices were published in *Out in Perth *Newspaper, The Melbourne *MCV *paper and *Lesbians on the Loose (LoTL)*, a nationally based lesbian magazine

• business-card sized 'flyers' were produced and sent to community and health centres across Australia and New Zealand as well as to gay and lesbian social and support groups

• study information was posted on a number of email lists including (but not limited to): Gay Dads Australia (and their state-based e-lists); Rainbow Families Council; ACT Queer; Australian Lesbian Medical Association; Gay and Lesbian Researchers; New Zealand GLBT Research Network; Auckland Gay and Lesbian Welfare; New Zealand Families; Gay and Lesbian Line; HOT News (South Australia); Pride Western Australia; Gay and Lesbian Equality Inc. Western Australia

• informal email publicity was generated by sending emails to personal networks and asking people to pass it on to friends and colleagues.

Some of the recruitment strategies used in 2008 have been repeated in 2009/2010 to attract more participants, with a particular emphasis on posting advertisements on relevant email lists and electronic networks. Advertisements calling for more participants have also appeared in an electronic newsletter sent to current participants and on the WLP project website. All advertising invites potential participants to visit the self-complete survey online or, in the case of electronic advertising, to click on a link which redirects people to the survey website.

### Data collection procedure and survey instrument

#### Self-report survey (on-line)

The 2008, 2010 and 2012 surveys will be completed online. For the Wave One survey (2008), a survey-hosting company set up the questionnaire online and collected and maintained the data on a secure server that is accessible only to the researchers via a password.

The Wave One questionnaire contained over 100 items and took approximately 30 to 60 minutes to complete. The electronic format allowed only relevant questions to be asked of each individual respondent. For example, questions about current relationships did not appear if a respondent noted in a previous question that they were currently single.

At the end of the survey, participants were asked if they would be willing to participate in future research and, if yes, to provide their contact details. It was made clear to respondents that this option was voluntary and that they could remain anonymous and only participate in Wave One of the WLP study.

The survey covered a range of topics and included a number of questions that matched those of other large Australian studies to enable comparison between the WLP sample and other, largely heterosexual, samples. Items in the WLP survey were grouped as follows (see also Table 1):

#### Socio-demographic variables

The survey included a set of demographic questions including the gender, sexuality, age, place of residence, education, occupation, cultural/ethnic background, languages spoken and income of respondents and, if relevant, their partner. If respondents indicated they were currently in a relationship they were asked questions about their relationship including length of time together and length of time cohabiting. They were also asked whether they currently had a parenting arrangement with anyone besides their partner (such as a donor, ex-partner and so forth). One open ended question asked respondents to describe their current family structure.

#### Parental status

Respondents were asked about the methods used for conception with each of their children, and their relationship status at the time of conception. Options for fostering, adoption or other permanent care arrangements were included in this.

#### Household division of labour

A set of questions about division of labour in the household was adapted from the 'Negotiating the Life Course' (NLC) study, a longitudinal study of Australian couples undertaken by the Australian Demographic and Social Research Institute at the Australian National University and the School of Social Sciences at the University of Queensland. The NLC survey uses a Computer Assisted Telephone Interview (CATI) method to collect information from 1500 Australian respondents every three years. The first wave of the NLC was conducted in 1996 and 1997, with three subsequent waves being conducted in 2000, 2003 and 2006 [50,51].

NLC questions used in the WLP survey relate to the allocation of time to both household and childcare tasks undertaken by each partner. Respondents were asked to indicate who in the household usually undertook a specific set of 17 different tasks: repairing things around the home, making arrangements to have repairs done, doing the dishes, preparing breakfast, preparing the evening meal, cleaning the house and vacuuming, doing the laundry, doing the ironing, cleaning the bathroom and toilet, caring for pets, taking out rubbish, shopping for food and other essentials, mowing the lawn, taking care of the garden, driving the car when going somewhere together, organising your social life and keeping in touch with relatives or friends. In relation to childcare responsibilities, respondents were asked about six different tasks: helping with homework, listening to problems, taking children to activities/appointments, playing with them, bathing and dressing, getting them to bed. Since only one partner responded to the survey, it relied on the reports of one individual to assess the way in which tasks are allocated. This may be a limitation of the data as it is not clear how reliable one person's estimates of the household division of labour may be [51].

#### Family and community life

A number of questions about family and community connection as well as health and wellbeing were adapted for the WLP survey from the Longitudinal Study of Australian Children (LSAC). LSAC follows two cohorts of children aged 0-1 years (cohort 1) and 1-5 years (cohort 2). The study will run for six years, with Wave 1 taking place in 2003, Wave 2 in 2006, Wave 3 in 2008 and Wave 4 in 2010. A total, of 10,090 participated in Wave 1 of the study, approximately 5000 in each cohort. Study informants include the child (when of an appropriate age) and their parents, carers and teachers [52]. The following questions were adapted from the parents' questionnaires used in LSAC for the WLP (Wave One) survey.

• ***Social networks (help and support): ***respondents were asked to whom they can turn for information about parenting, emotional support, financial assistance, practical help and information as well as whether they feel they get enough support. These questions were adopted and modified for the LSAC survey from the Australian Life Course survey [53]. Extra questions were included in the WLP survey about respondents' perception of the support they receive from their same-sex partner's family.

• ***Community connection: ***five questions were asked about respondents' perceptions of their local community including how they feel about the neighbourhood, whether neighbours can be trusted and whether they perceive the neighbourhood to be a good place to bring up children. These questions were originally from the Canadian Longitudinal Study of Children and Youth [54] and the World Values Survey [55]. WLP respondents were also asked to indicate whether they are involved in locally based organisations as a participant or volunteer (adopted from the Canadian National Longitudinal Study of Children and Youth)[54]. The items on volunteering were expanded for the WLP study from those used in LSAC to differentiate between the respondent's participation in local organisations, their children's participation and their partner's. WLP respondents were also asked questions about: their level of engagement with the lesbian and gay community; how open they are about their sexuality in relation to a range of mainstream community organisations such as schools, childcare centres, health services and so forth; and whether they had experienced discrimination in any of these organisations.

• ***Childcare: ***WLP included four questions which asked respondents about the type of childcare they currently used and their reasons for choosing their current childcare arrangements. The LSAC version of these questions was derived from the Australian Bureau of Statistics [56] and the National Institute of Child Health and Human Development study of Early Childcare and Youth Development [57]. Extra questions were added to the WLP survey asking respondents whether concerns about homophobia influenced their decisions about childcare options. For example, participants were asked how important it was to them to find a lesbian and gay friendly childcare centre.

• ***Work and family***: one question asking respondents about perceived control within their paid workplace was used for the LSAC survey from the Household, Income and Labour Dynamics in Australia (HILDA) study [58]. Another 11 items were adopted from the 13-item 'Work and Family Strains and Gains" scale [59], which asked respondents how they feel about the balance they achieve between work and parenting responsibilities. Within this, three subscales looked at: work having a negative impact on family time; work having a positive impact on family time; and respondent's sense of self, competence and wellbeing derived from work. WLP study respondents were also asked to describe qualitatively the way in which they organise household and work responsibilities and their reasons for organising it as such.

• ***Parenting and relationship conflict: ***the WLP survey incorporated a five-item subscale from the Quality of Co-parental Interaction Scale that was used in the LSAC survey to assess conflict between separated/divorced spouses over parenting issues [60].

• ***Partner support: ***the WLP survey included three items that were from Ahron's (1981) Quality of Co-parental Interaction Scale [60] to measure the extent to which respondents feel their partner is a resource/support in raising children, how often they are a resource/support to their partner and how well their partner meets their needs.

• ***Relationship satisfaction: ***six items were taken from the LSAC survey regarding the extent to which respondents feel that their partner meets their needs, perceived quality of relationship compared to others, extent to which their relationship has met respondents' expectations, problems within the relationship and feelings for their partner. These were originally derived from the Relationship Assessment Scale [61]. A further item was also adopted from the LSAC study which asks about respondents to rate their level of happiness with their relationship. This was derived from an abbreviated version of the Spanier Dyadic Adjustment Scale [62].

• ***Health and wellbeing***: the WLP survey included the Kessler (K6) screening scale to measure parental psychological distress over the last four weeks across six items on a five-point Likert scale ranging from 'all of the time' to 'none of the time' [63] as well as a two-item measure of parental depression over the last year/two years that was derived for the LSAC study from the Family Psychosocial Screen, adapted from RAND Corporation's eight-item Screening Instrument for Depressive Disorders [64]. WLP also included questions on participants' recent use of health services and whether they had ever accessed psychological/counseling support for relationship issues.

#### Relationship and life satisfaction

A set of questions about respondents' satisfaction with life was adapted from the Australian Longitudinal Study on Women's Health (Women's Health Australia). The Women's Health Australia study is a longitudinal population-based survey, which examines the health of more than 40,000 Australian women over a 20 year period. The study began in 1996 [65]. A total of eight questions from the WHA study were adopted for the WLP survey asking respondents about their level of satisfaction with: work, career and study achievements; family relationships; partner relationships; friendships; social activities; and parenthood. Satisfaction was measured using a 4-point Likert scale ('very satisfied' to 'very dissatisfied').

#### Formalising relationships

Respondents in a relationship were asked if they had ever undertaken a formal or informal commitment ceremony, civil union or marriage in areas where this is legal and if they felt the current legal status of same-sex couples made them feel more vulnerable as a parent.

#### Experience of family

A number of open-ended questions were included in the WLP survey which asked respondents to examine issues including: the way in which their relationship with their extended family changed when they became a parent, challenges and difficulties in their family life, positive aspects of their family life and the impact of the legal status of gay and lesbian relationships on their families.

### Qualitative sub-studies

Two sub-studies will be conducted as part of the WLP study. In the first substudy, all participants will be invited to participate. Those who nominate to be involved will be selected on the basis of their location of residence, gender and age of children. These interviews will focus on participants' perceptions of personal, relationship and community/social factors that support resiliency in their family. In families where there are two parents, both parents will be invited to attend an interview.

The second sub-study on the experience of parental separation in same-sex families will include a sample of families where the parents have separated during the study period. An invitation to participate in the study will be sent to all participants explaining the criteria for the sub-study and requesting eligible people to volunteer. We aim to make contact with both partners and interview them separately.

The interviews will be conducted in Years 3 and 4 of the study (2012-2013). Respondents from at least four Australian states or territories (Victoria, NSW, Queensland and the ACT) will be included in the sub-studies and they will include people from both metropolitan and rural/remote areas. Interviews will be face to face or via telephone for some individuals who live in more remote locations. Approximately 20 families from each subgroup will be interviewed (40 families in total).

Interview data will build on the information gained though Waves 1 and 2 of the study by allowing couples to reflect on factors that are most strongly associated with resilience for them personally (substudy one and two), factors that were associated with their relationship breakdown (substudy two) and the experiences of separation and loss of relationship (substudy two). The interviews will also involve discussion of the range of social supports that same-sex couples utilised before, during and after separation including health, counselling or legal services, and the extent to which these services met their needs (substudy two). Interviews will be semi-structured, enabling preliminary survey findings to be tested and explored with interview participants, while also leaving room for participants to identify other issues and topics of relevance.

#### Ethics

The Faculty of Health Sciences, Human Ethics Committee, LaTrobe University has approved this research (FHE 08/33). The major ethical concern for the WLP study lies in protecting the confidentiality of respondents. It is necessary for identifying information about respondents (names and contact details) to be collected so that respondents can be invited to participate in future surveys and their responses can be matched across the three waves of the longitudinal study. In order to manage this, respondents' contact details are stored in a password protected electronic database that is separate to the main dataset. Their responses can be matched to the main dataset (which is also password protected) via a unique ID code. Only two of the researchers have access to this information.

## Discussion

The Work, Love, Play study is the first large-scale study of same-sex parented families conducted in Australia and New Zealand. Its significance lies in the major contribution it will make to national and international literature on family resiliency. Further, as there have been no previous studies on the experience of separation among same-sex parented families, this research will fill a major gap in the international literature on this topic. The study will also provide an important contribution to the evidence base regarding the health and wellbeing of same-sex attracted parents and their children.

Other strengths of the study include the large sample size compared to previous studies of this population and inclusion of both lesbian and gay male parents. There is only a limited number of published studies of gay male parents [66-69] and previous studies of lesbian parents tend to be qualitative studies involving small samples [4,8,9,12,70-73].

The mixed method (qualitative and quantitative) approach to this study is appropriate for studying the concept of resilience in same-sex parented families. As there has been little research on this topic, the collection of qualitative data will enable further exploration and clarification of the major themes emerging from Waves One and Two of the study.

The prospective, longitudinal design of the study creates an opportunity to explore the experience of separation among a sample of same-sex parents, including examination of factors leading up to separation and parental wellbeing both pre and post separation. Additionally, the design enables examination of family wellbeing over time in Australian state and territories where the laws relating to same-sex parenting have changed since Wave One. For example, there are currently 83 respondents from Victoria, Australia who have agreed to participate in future waves of the study. New laws which enable two mothers to appear on a child's birth certificate and provide greater access to reproductive technology for prospective lesbian parents came into effect in this State in January 2010.

There are some limitations to the WLP study. There is no sampling frame of lesbian and gay parents from which to draw a random selection of participants for this study. The non-probability sampling methods used in this study potentially creates some bias in the sample as those more connected to social and support networks are more likely to have been exposed to information about the study. Unfortunately this means people who are more socially isolated and/or who have poorer mental health may be under-represented in the sample. The on-line survey method also means it is not possible to determine a response rate as it is not known how many people saw information about the study and declined to participate.

The current Wave One sample in this study had notably high levels of education. This is not uncommon. Most Australian studies of gay, lesbian, bisexual and transgender (GLBT) populations involve samples who have higher average education levels than the general population [14,28,74]. It is unclear whether this reflects an actual higher level of education among Australians who openly identify as same-sex attracted or whether people from lower socio-economic backgrounds are less likely to participate in research, particularly internet based research. Similarly, there was a low representation of people from non-English speaking backgrounds in the sample. Again, this is not uncommon in Australian research that is not specifically targeted toward a cultural group [74]. The ethnic diversity in both Australia and New Zealand makes it difficult to represent adequately the experiences of all people from such a large range of cultural backgrounds. Without extensive targeting of particular cultural groups it is difficult to achieve adequate numbers to ensure representation within the overall sample. It should be noted that the experiences of same-sex attracted parents may differ across different social and cultural groups in Australia and New Zealand.

## Competing interests

The authors declare that they have no competing interests.

## Authors' contributions

JP contributed to the design of the study and survey instrument, implemented Wave One of the study and drafted this manuscript. All other authors contributed to the design of the study and survey instrument, assisted with implementation of the study and assisted to edit this manuscript. All authors read and approved the final manuscript.

## Pre-publication history

The pre-publication history for this paper can be accessed here:

http://www.biomedcentral.com/1471-2458/10/115/prepub
